# Supplementary material for: Shining light on vitamin C deficiency and scurvy in Canada: A scoping review protocol of risk profiles, health outcomes, and interventions
Source: PLoS One. 2026 Mar 9;21(3):e0336664. doi: 10.1371/journal.pone.0336664 (PMC12970891; doi:10.1371/journal.pone.0336664)
Supplement: S1 Appendix — (DOCX) [file pone.0336664.s001.docx]

**APPENDIX**

**Eligibility Criteria for Literature Screening in Covidence**

**Population (Inclusion)**

- Indigenous and non-indigenous populations in Canada.

**Population (exclusion)**

- Populations outside Canada.

**Intervention / Exposure (Inclusion)**

- Any intervention, program, or policy aimed at addressing scurvy and Vitamin C deficiency in Canada. Example dietary changes, supplementation, Nutritional education, or health promotion.
- Health system responses to scurvy and vitamin C deficiency in Canada.
- Risk/predisposing factors to scurvy and vitamin C deficiency among Canadian populations.
- Studies utilizing geospatial science and tools (geospatial informatics systems and geodemographic segmentation methodology) to study the incidence/prevalence of scurvy and vitamin C deficiency and predisposing factors in the Canadian context.
- Social Lifestyle

**Intervention (Exclusion)**

- Interventions unrelated to scurvy or Vitamin C deficiency.
- Intervention addressing scurvy and vitamin C deficiency outside Canada.
- Studies utilizing geospatial science and tools (geospatial informatics systems and geodemographic segmentation methodology) to study the incidence/prevalence of scurvy and vitamin C deficiency and predisposing factors outside the Canadian context.

**Outcome (Inclusion)**

- Prevalence and incidence of scurvy and vitamin C deficiency in Canada.
- Mortality and Morbidity rates of scurvy and vitamin C deficiency in Canada.
- Public health implications of scurvy and vitamin C deficiency in Canada.
- Other health outcomes of scurvy and vitamin deficiency in Canada.
- Outcomes pointing to a regional association (context of geographic location) with scurvy and an increased risk of insufficient vitamin C intake in the Canadian context.

**Outcome (Exclusion)**

- Outcomes not related to scurvy or Vitamin C deficiency.
- Biochemical studies without human population data.
- Outcomes related to scurvy or vitamin C deficiency outside the Canadian context.

**Study Characteristics (Inclusion)**

- Literature published in English.
- Human studies.
- Studies published from January 2000
- Peer-reviewed

**Study Characteristics (Exclusion)**

- Non-English Literature.
- Conference abstracts without full papers.
- Non-human studies.
- Studies published before January 2000

**Other (Inclusion)**

- All empirical studies: qualitative, quantitative, mixed methods
- Reviews, program reports, case reports/case series, surveillance reports, government documents

**Other (Exclusion)**

- Editorials, opinion pieces, and news articles without primary or secondary data
- Animal studies
- Preprints
